# Hepatoprotective effect of crude polysaccharide isolated from *Lycium barbarum* L. against alcohol‐induced oxidative damage involves Nrf2 signaling

**DOI:** 10.1002/fsn3.1942

**Published:** 2020-10-29

**Authors:** Han Wang, Yongsheng Li, Jianfei Liu, Duolong Di, Yewei Liu, Jianteng Wei

**Affiliations:** ^1^ Key Laboratory of Chemistry of Northwestern Plant Resources Lanzhou Institute of Chemical Physics Chinese Academy of Sciences (CAS) Lanzhou China; ^2^ Center of Resource Chemical and New Material Qingdao China; ^3^ School of Public Health Lanzhou University Lanzhou China; ^4^ University of Chinese Academy of Sciences Lanzhou China

**Keywords:** alcohol, apoptosis, *Lycium barbarum* L., oxidative damage, polysaccharide

## Abstract

In the present work, we investigated the effect of *Lycium barbarum* L. polysaccharides (LBPs) on L‐02 cells exposed to alcohol exploring the potential molecular mechanisms. Our results suggested that LBPs significantly prevented alcohol‐induced hepatotoxicity with dose‐dependent effect, indicated by both cell viability and diagnostic indicators of liver damage. Moreover, alcohol induced excessive oxidative stress, as evidenced by an increase of the malondialdehyde level and reactive oxygen species production, while reducing antioxidant enzymes (T‐SOD, CAT, and GPx) in liver, were inhibited by administration of LBPs. Furthermore, LBPs reversed the cell apoptosis and increased the mitochondrial membrane potential in alcohol‐treated liver cell. Studies of underlying mechanisms revealed that LBPs increased expression levels of Nrf2 expression, which in turn blocked proapoptotic signaling events, restoring the balance between proapoptotic Bax and antiapoptotic Bcl‐2 proteins, suppressing activities of cytochrome C (Cyto c), caspase‐3, and caspase‐9 in L‐02 cells stimulation by ethanol. In general, the results showed that the inhibition of alcohol‐caused liver damage by LBPs is due at least in part to its antioxidant and antiapoptosis activity via Nrf2 signaling pathway.

## Graphical abstract

In the present work, we investigated the effect of polysaccharides from *Lycium barbarum* (LBPs) on L‐02 cells exposed to ethanol exploring the potential molecular mechanisms. Our results suggested that the inhibition of ethanol‐induced apoptosis by LBPs is due at least in part to its antioxidant and antiapoptosis activity via Nrf2 signaling pathway.

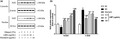

## 1 INTRODUCTION

Alcoholic liver disease (ALD) has been an important health and social hazard in the Western world, and its prevention and cure has become an important medical task (Kerr et al., 2000). As an economy develops, the morbidity and mortality of ALD has risen year by year in China recently (Zhu et al., 2015). The cancer probability increased with patient's age, disease course and ALD severity, and even life‐threatening. It has since been proven that one of the ALD pathological mechanisms is generation of oxidative alcohol metabolites (Seth et al., 2008; Song et al., 2004). Many research findings suggested that alcohol could act as a hepatotoxin that interferes with mitochondrial function in hepatocytes, leading to an accumulation of lipid and cell death (Cohen et al., 2009; Mandrekar & Szabo, 2009). Accordingly, therapies against oxidants and cell death could effectively relieve the progression of alcohol‐induced liver injury. The natural antioxidants may act an effective protection against oxidative stress‐related liver pathologies due to particular synergisms (Balunas & Kinghorn, 2005; Huang et al., 2012).

Historically consumed as a “super food” among the population, *Lycium barbarum* has been widely applied as pharmaceuticals, health food, and functional food for centuries (Chang & So, 2008; Kim et al., 1997). In “Ben Cao Gang mu,” it is recorded that Chinese wolfberry has an action on liver and kidney protection, immunity‐boosting properties, antiaging, profits lung, and eyesight effects. Particularly, the bio‐efficiency of *Lycium barbarum* arises from polysaccharide complex (LBPs), the liquid fraction extraction in a number of studies (Li et al., 2007; Xin et al., 2011). As a potent antioxidant, the primary experiment has revealed that LBPs has satisfactory actions of regulating antioxidant biomarkers and protecting the body from damage related to oxidative stress (Amagase et al., 2009; Wu et al., 2010; Xin et al., 2011). In addition, LBPs was reported to attenuate oxidative stress after hepatotoxin, associated with hepatocellular proliferation, apoptosis, and lipid production (Cheng & Hong, 2011; Jia et al., 2012; Yang et al., 2017). Despite those pharmacological benefits, the hepatoprotective activity and relevant mechanisms of LBPs against alcohol‐induced damage were not studied in detail previously.

Thus, the present study was designed to examine the protective ability of *Lycium barbarum* L. polysaccharides (LBPs) extract against alcohol‐induced hepatotoxicity in L‐02 liver cell, thus to clarify the mechanisms of protective effects on oxidative stress and its clinical significance.

## 2 MATERIAL AND METHODS

### 2.1 Materials and regents

RMPI 1640 medium and fetal bovine serum were obtained from Hyclone (Thermo Fisher). Dimethyl sulfoxide (DMSO), 3‐(4, 5‐dimethylthiazol‐2‐yl)‐2, 5‐diphenyltetrazolium bromide (MTT), phosphate buffer saline (PBS), reactive oxygen species (ROS) detection kit, lactate dehydrogenase (LDH) kit, mitochondrial membrane potential (MMP) assay kit with JC‐1, RIPA buffer, and BCA protein quantitation kit were obtained from Solabio. Alanine aminotransferase (ALT), aspartate aminotransferase (AST), total antioxidant capacity (T‐AOC), superoxidase dismutase (SOD), catalase (CAT) as well as glutathione peroxidase (GSH‐PX), and malondialdehyde (MDA) assay kits were purchased from Nanjing Jiancheng bioengineering institute. Annexin V‐FITC apoptosis detection kit, phosphatase inhibitor cocktail, nuclear protein and cytoplasmic protein extraction kit were supplied by BestBio. Anti‐β‐actin and antiproliferating cell nuclear antigen (PCNA) antibodies were provided by ABclonal. Antinuclear factor erythroid‐2‐related factor 2 (Nrf2), anti‐B‐cell lymphoma‐2 (Bcl‐2), anti‐BCL2‐Associated X (Bax), anti‐Cytochrome C (Cyt c), anti‐Caspase‐9, and anti‐Caspase‐3 were supplied by ABCAM. Anti‐Bcl‐xL/Bcl‐2 associated death promoter (Bad) and anti‐p‐Bad were purchased from Cell Signaling Technology. Anti‐Rabbit Detection Module for Wes and 12–230 kDa Wes Separation Module were purchased from ProteinSimple.

### 2.2 Preparation of LBPs

Crude polysaccharide of *Lycium barbarum* L. was prepared by an integrated membrane technology. Briefly, 200 g the fruiting bodies of *Lycium barbarum* L. were treated with high‐speed shearing at 60°C in ratio of material to liquid 1:12. After precipitated with 70% alcohol, the pretreated extracting solution was filtered with a 5,000 Da membrane. The resulting sample was collected and lyophilized as crude LBPs. The content of the polysaccharides was measured by school of public health, Lanzhou University, in the method of phenol‐vitriol according to the Chinese Pharmacopoeia, and the yield of LBPs was 60.50% (w/w). The LBPs was dissolved in distilled water and filtered and then stored in 4°C before use.

### 2.3 Cell culture

The human L‐02 liver cell line was acquired from institute of cell biology, Chinese academy sciences. Cells from exponential phase were cultured in serum‐containing RPMI 1640 medium supplemented with 1% penicillin/streptomycin in humidified atmosphere with 5% CO_2_ at 37°C.

### 2.4 Cell viability assay

Cells (1.2 × 10^5^/ml) were placed in 12‐well plates and incubated for 24 hr. The cells were then treated with 5% alcohol in triplicate. After 4 hr exposure, cells of normal control (NC) and model control (MC) received fresh medium. Silibinin (100 μg/ml) was used as positive control. Other cells were stimulated in the presence of various concentrations of LBPs (12.5, 25, 100 μg/ml) for 24 hr. Then, 20 μl MTT to a final concentration of 5 mg/ml was added for further 2 hr. Subsequently, purple‐blue formazan precipitate was dissolved with DMSO (150 μl), and the optical densities (OD) at 495 nm were measured by a microplate reader (DNM‐9602G, Perlong).

### 2.5 Determination of ALT, AST, and LDH enzyme activities

The cell culture supernatant was harvested. The ATL, AST, and LDH levels were assayed with commercially available kits according to the manufacturer's protocols.

### 2.6 Antioxidase activity assay

The cells were scraped and broken into homogenates in cold PBS with ultrasonic cell crusher. The supernatants in cell lysates were collected for determination of intracellular activities of MDA, SOD, CAT, and GSH‐Px with commercially available assay kits by spectrophotometer.

### 2.7 Measurement of ROS accumulation

Intracellular ROS generation was measured by the CM‐DCFH oxidized product, CM‐DCF fluorescence. After incubation, cells were trypsinized and rinsed with ice‐cold PBS. DCFH‐DA (10 μM) was mixed and incubated at 37°C in dark. Following staining for 20 min, cells were washed in PBS and analyzed by confocal laser scanning microscopy (FV1200, Olympus). The relative fluorescent intensity at 488 nm excitation and 525 nm emission wavelengths was examined under fluorescence spectrophotometer (Fluoromax‐4P, Horiba).

### 2.8 Cell staining and cell apoptosis detection

The cells were scraped after LBPs treated and stained with Hoechst 33258 (10 μg/ml), a cell‐permeable DNA dye, at 37°C for 30 min. The fresh medium was applied to remove the residual dye. The change of cell morphology was observed through confocal laser scanning microscopy with 352 nm/461 nm.

The influence of LBPs on the apoptotic rate of L‐02 cells induced by alcohol was analyzed with Annexin V‐FITC apoptosis detection kit. The cells were detached and collected by centrifuging at 600 *g* for 5 min. After washed with PBS, a total of 400 μl 1× Annexin V binding buffer and 5 μl Annexin V‐FITC of staining solution were added to the cell suspension for 15 min at 4°C. Subsequently, the 10 μl PI was mixed and incubated for 5 min in the dark. The quantification of apoptosis cells was detected at the end of action by flow cytometry (LSRFortessa, BD).

### 2.9 Measurement of MMP

MMP was observed using the JC‐1 fluorescent probe by confocal laser scanning microscopy in accordance with the instructions. Following 20 min staining with JC‐1 working solution at 37°C in dark, the fluorescent intensities were monitored the dual emissions from monomers (green fluorescence, Ex/Em: 485 nm/535 nm) and aggregates (red fluorescence, Ex/Em: 535 nm/595 nm). The relative amounts of green/red fluorescence were performed using flow cytometry.

### 2.10 Western blot analysis

The cells were scraped and washed with PBS and homogenized in RIPA buffer with 1% phosphatase inhibitors and 1% PMSF for 25 min on ice. Cell homogenates were centrifugation at 12,000 *g* for 15 min at 4°C. The cytoplasmic and nuclear proteins were obtained using nuclear/cytoplasmic isolation kit according to manufacturer's protocol. Protein concentrations of cell extracts were determined by BCA protein assay kit. Wes analysis was performed on a Wes system according to the manufacturer's instructions. In brief, protein samples were diluted to an appropriate concentration in lysate containing PMSF (1%) and phosphatase inhibitor (1%) and then mixed with Loading Buffer and Sample Buffer. The mixture was heated at 95°C for 5 min and then cooling on ice. The samples, blocking reagent (antibody diluent), primary antibodies (1:50 dilution for p‐Bad, 1:100 dilution for β‐actin, Nrf2, Bad, Bcl‐2, Bax, Caspase‐3, Caspase‐9, Cyt‐C, PCNA), HRP‐conjugated secondary antibodies, and chemiluminescent substrate were dispensed into the plate. After plate loading, the separation electrophoresis and immunodetection steps take place in Wes, the fully automated capillary system, equipped with Compass for SW analysis software (ProteinSimple).

### 2.11 Statistical analysis

Experimental values were represented as mean ± standard deviation (*SD*). Data on significant difference between groups were analyzed using one‐way analysis of variance (ANOVA) by SPSS statistics version 19.0 (IBM SPSS Inc.). A value of *p* < .05 was considered statistically significant.

## 3 RESULTS

### 3.1 LBPs improve viability of alcohol‐treated L‐02 cells

**Figure 1:**
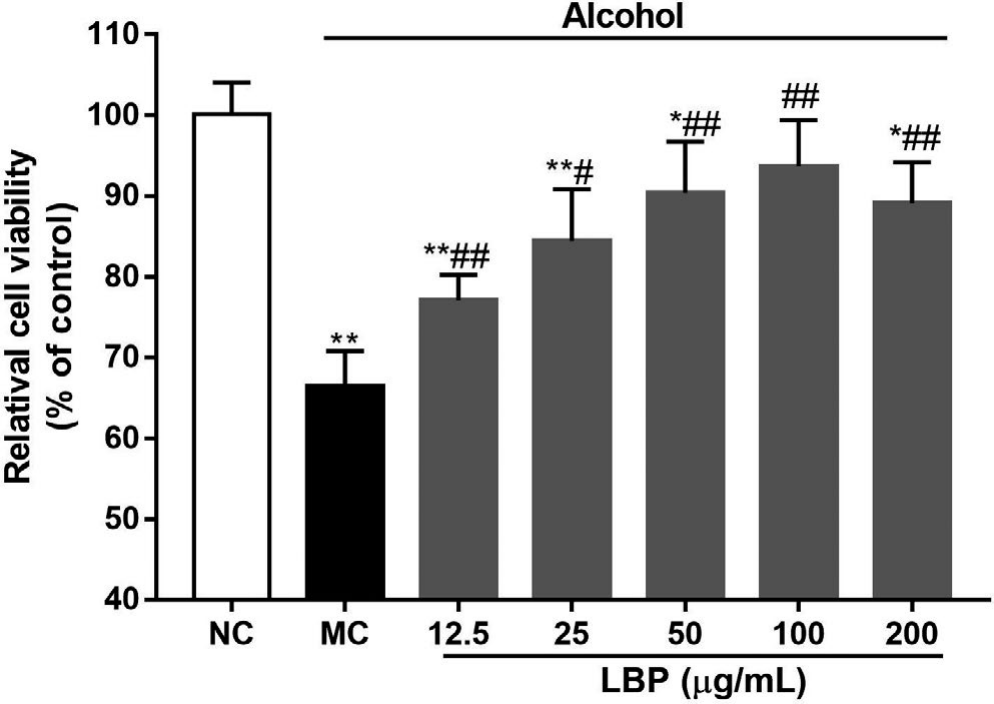
The effect of crude polysaccharide extracted from *Lycium barbarum* L. (LBPs) on cell viability in alcohol induced L‐02 cells (*n* = 6). Compared with the NC group *p < .05, **p < .01; compared with the MC group ^#^ *p* < .05, ^##^ *p* < .01

To determine whether LBPs can attenuate the toxicity of acute alcohol treatment, we measured the cell survival rates of the L‐02 cells after being pretreated with 5% alcohol for 4 hr. As shown in Figure 1, the incubation of L‐02 cells with 5% alcohol induced about 40.0% growth inhibition compared to the untreated controls (*p* < .01). In the range of 12.5–100 μg/ml, the addition of LBPs to the alcohol‐pretreated cells exerted a protective effect reducing cell growth inhibition. When the concentration of LBPs reached 100 μg/ml, cellular survival rate obviously rose (*p* < .01 vs. alcohol alone), which was near to baseline level of control cells. Therefore, the concentration of 12.5, 25, and 100 μg/ml was chosen for the further investigation.

### 3.2 LBPs ameliorate hepatic enzyme in alcohol‐treated L‐02 cells

**Figure 2:**
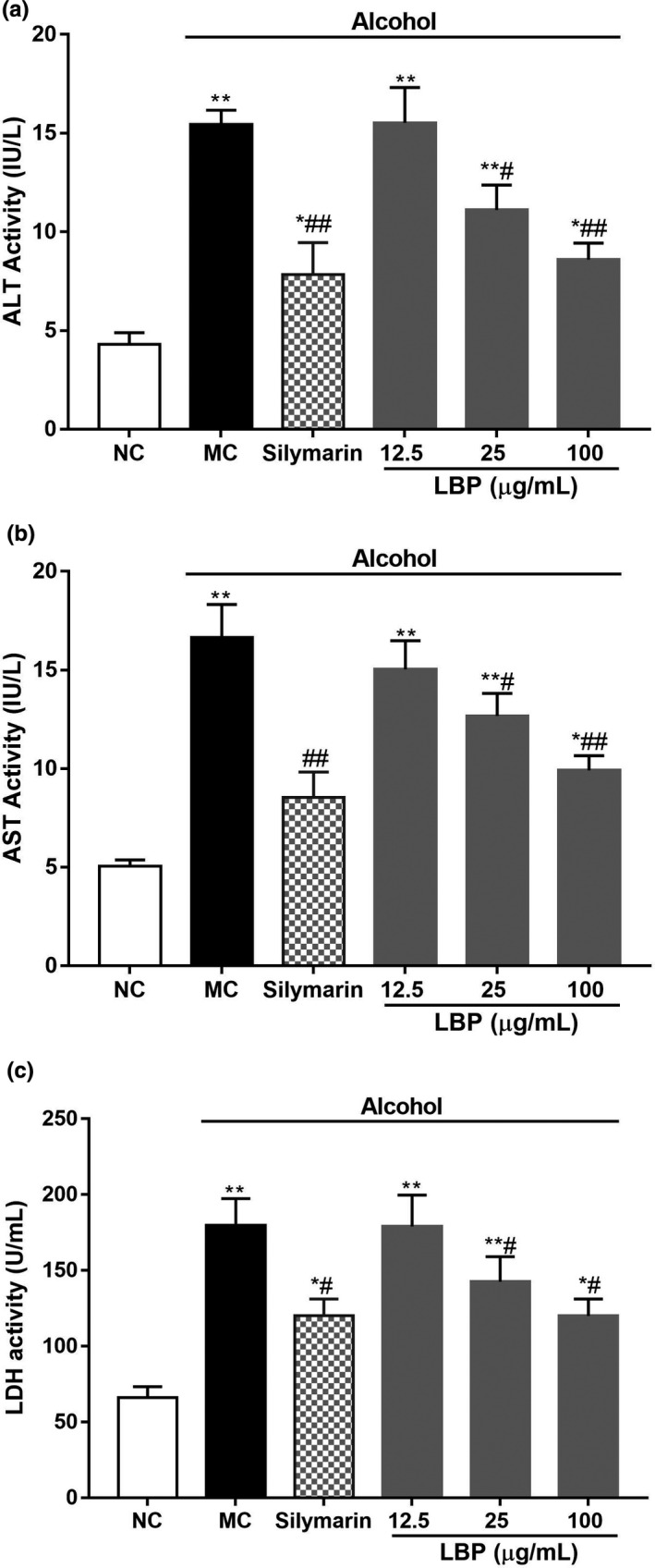
The effect of LBPs on alcohol‐induced hepatic enzyme release in alcohol induced L‐02 cells (a) ALT, (b) AST, (c) LDH (*n* = 6). Compared with the NC group *p < .05, **p < .01; compared with the MC group ^#^ *p* < .05, ^##^ *p* < .01

Moreover, administration of alcohol determined a significant enhancement in biomarkers of diseases hepatic damage, namely ALT, AST, and LDH in L‐02. In Figure 2a–c, the levels of ALT, AST, and LDH were significantly increased to 3.59‐, 3.28‐, and 2.72‐fold as compared to normal cells, respectively, (*p* < .01) while LBPs treatment provided dose‐dependent inhibition in this elevation.

### 3.3 LBPs repaired the activities of antioxidant enzymes in alcohol‐treated L‐02 cells

**Figure 3:**
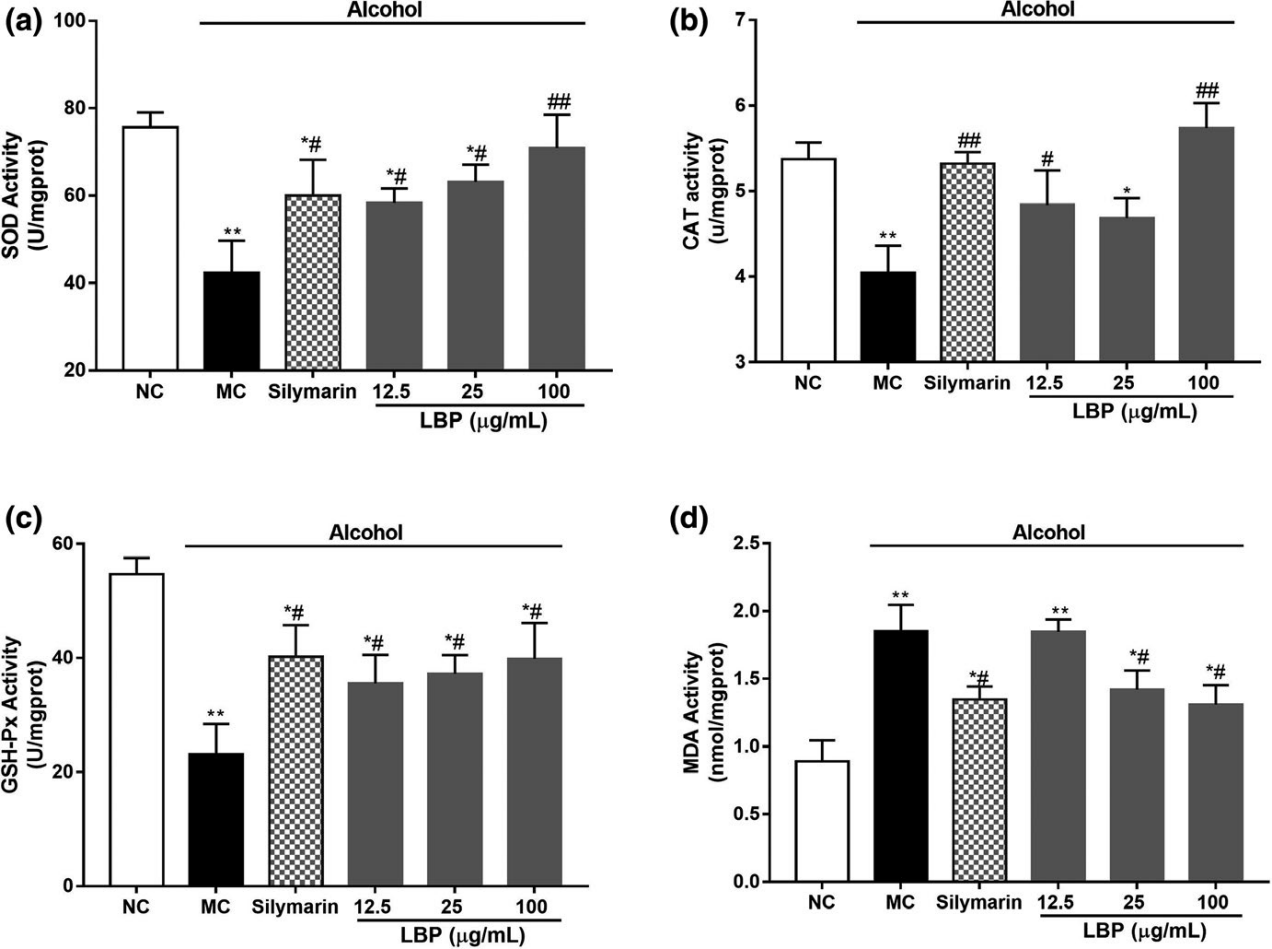
The effect of LBPs on the activities of antioxidant enzymes in alcohol‐treated L‐02 cells (a) SOD; (b) CAT; (c) GSH‐Px; (d) MDA (*n* = 6). Compared with the NC group *p < .05, **p < .01; compared with the MC group ^#^ *p* < .05, ^##^ *p* < .01

The activities of SOD, CAT, GSH‐Px, and MDA, which participate in intracellular redox balance, were measured by spectrophotometry. As shown in Figure 3a–c, SOD, CAT, and GSH‐Px activities were significantly decreased in alcohol‐treated cell by 44.0, 24.7, and 57.7% than those of the NC group, respectively (*p* < .01). For comparison, LBPs reversed the SOD, CAT, and GSH‐Px activities with dose‐dependent effect. On the other hand, alcohol treatment could cause an apparently increase in the accumulation of the marker for endogenous lipid peroxidation‐MDA, as compared with NC group (Figure 3d, *p* < .01). Nevertheless, LBPs at test concentration prevented the elevation of MDA significantly and dose‐dependently (*p* < .05 vs. alcohol alone).

### 3.4 LBPs attenuated alcohol‐induced oxidative stress in alcohol‐treated L‐02 cells

**Figure 4:**
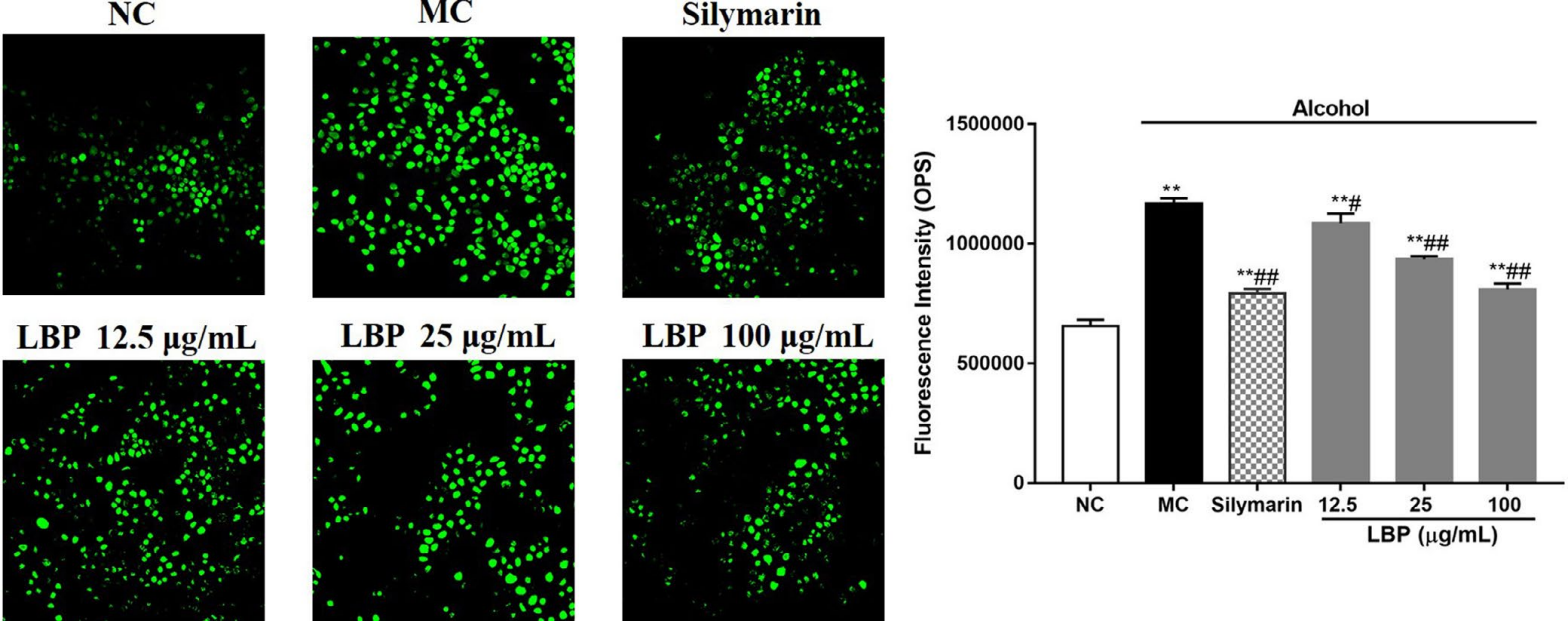
The effect of LBPs on the level of ROS in alcohol‐treated L‐02 cells (*n* = 6) (a) fluorescence quantitative analysis on the level of ROS; (b) The histogram of fluorescence intensity. Compared with the NC group *p < .05, **p < .01; compared with the MC group ^#^ *p* < .05, ^##^ *p* < .01

In order to further determine the protection of LBPs on oxidative damage in L‐02 cells, we assessed the state of ROS by examining the fluorescent intensity of DCFH‐DA‐incubated cells. The cells intoxicated with alcohol exerted a significant change (*p* < .01) in the ROS levels, which increased by 1.78‐fold compared to the NC group, as shown in Figure 4b. The LBPs treatment demonstrated protection against alcohol‐induced injury in hepatic pathophysiology by lowering the elevation of the ROS level to the normal level, further confirming the antioxidant ability of LBPs.

### 3.5 LBPs suppressed alcohol‐induced disruption of MMP

**Figure 5:**
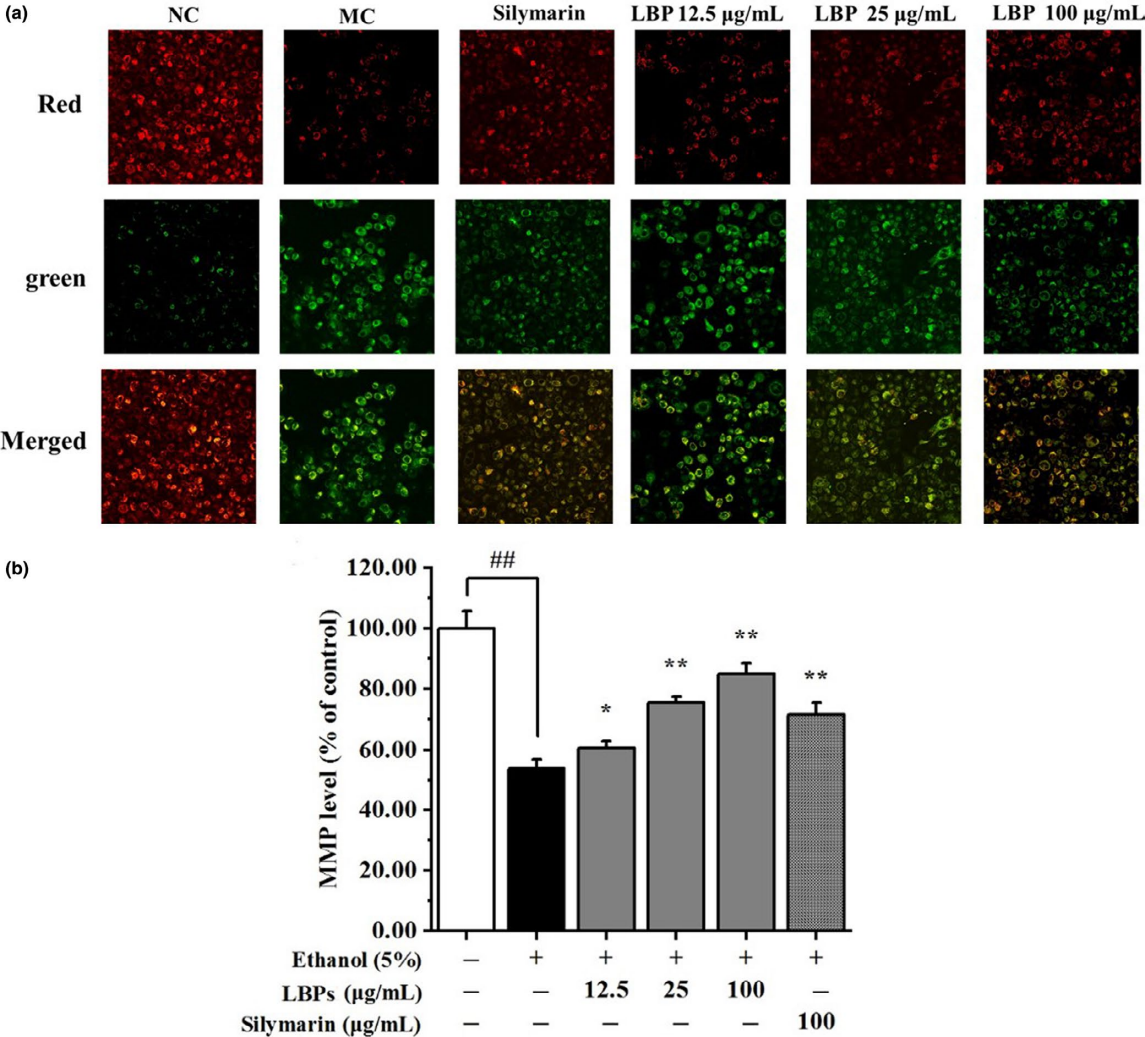
The effect of LBPs on the MMP in alcohol‐treated L‐02 cells (*n* = 3). (a) The effect of LBPs on MMP of apoptotic cells; (b) The histogram of relative MMP level. Compared with the NC group *p < .05, **p < .01; compared with the MC group ^#^ *p* < .05, ^##^ *p* < .01

The ROS generation and oxidative stress has been the cause of mitochondria depolarization, resulting in cell death (Tian et al., 2008). Thus, we examined whether LBPs could attenuate the mitochondrion‐dependent cell apoptosis in L‐02 cells. Figure 5a showed the change of MMP by confocal laser scanning microscopy. The results suggested that alcohol treatment significantly reduced red fluorescence (JC‐1 polymer), which correlated with polarization of the mitochondrial membrane. An increase of green fluorescence (JC‐1 monomer) was monitored in the presence of alcohol, indicated depolarization of mitochondrial membrane. The comparison of the relative amounts of green/red fluorescence in Figure 5b displayed that coadministration of LBPs reversed MMP greatly in a dose‐dependent manner (*p* < .05). LBPs ameliorate mitochondrial damage in alcohol‐treated hepatocyte.

### 3.6 LBPs protected L‐02 hepatocyte from alcohol‐induced cell death

**Figure 6:**
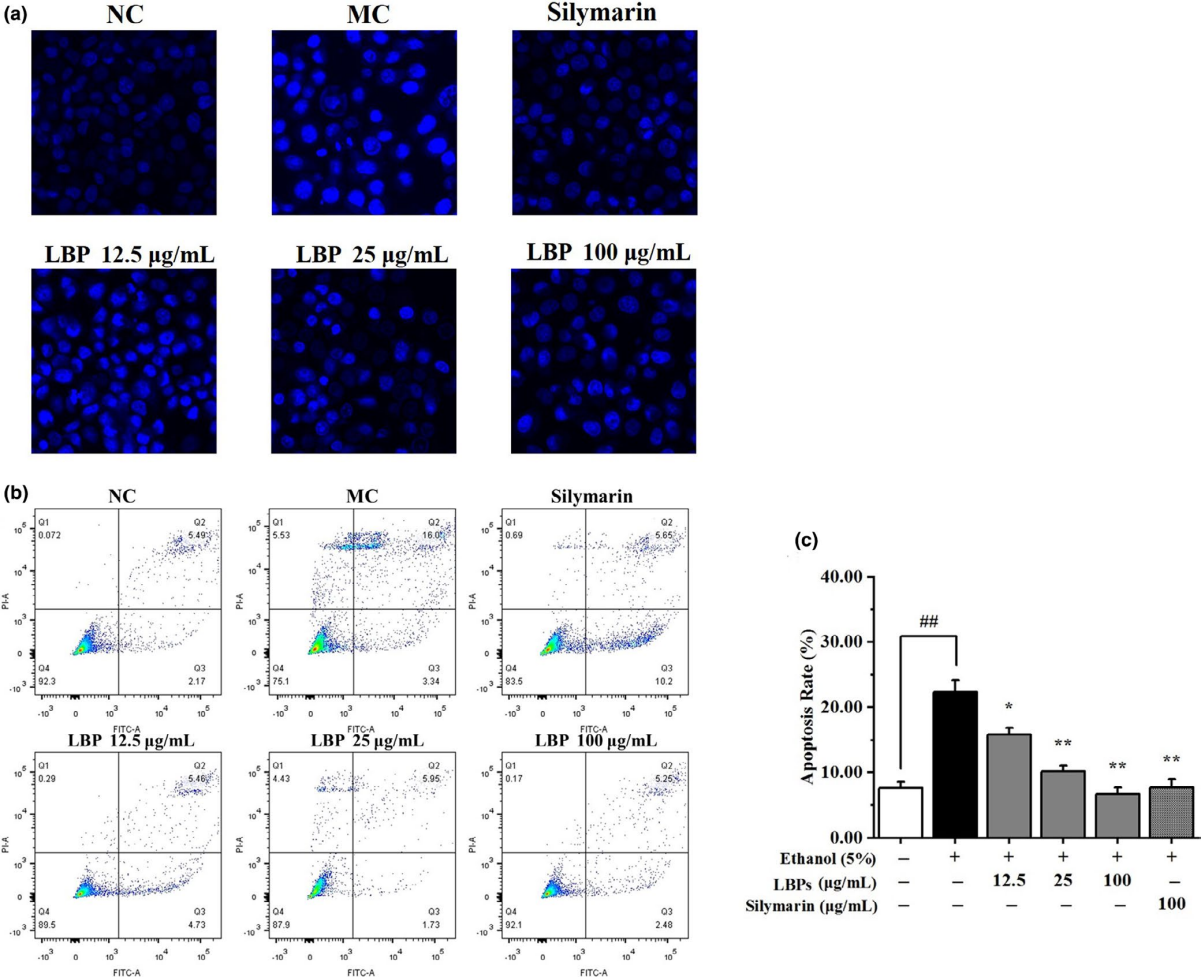
The effect of LBPs on the cell apoptosis in alcohol‐treated L‐02 cells (*n* = 3). (a) The effect of LBPs on the nuclei of apoptotic cells stained by Hoechst33258; (b) The percentage of apoptotic cells was measured by flow cytometry; (c) The histogram of apoptotic rate. Compared with the NC group *p < .05, **p < .01; compared with the MC group ^#^ *p* < .05, ^##^ *p* < .01

Based on the above results, it was hypothesized that LBPs could attenuate alcohol‐induced cell apoptosis. Thus, the nuclei of apoptotic cells stained by Hoechst33258 were observed by fluorescence microscopy to further examination. In Figure 6a, the cells in the alcohol groups were consonant with characteristic morphological changes of apoptosis, including cell contraction, pyknosis, and karyorrhexis. More interestingly, LBPs provided most obvious promotion on restoration, which were almost recovered to normal scale.

Moreover, Annexin V‐FITC/PI double staining and flow cytometry was applied to further investigate alcohol‐induced apoptosis (Figure 6b). Compared with those cells in NC group, the apoptosis percentage of L‐02 cells treated with alcohol was significantly increased from 7.66% to 19.34% (*p* < .01). The combined treatment of LBPs and alcohol was found to be effective at lowering the apoptotic rate of the cells dose‐dependently. The cellular state of high dose of LBPs (100 μg/ml) was even returned to its normal level. These experimental results further underscored the excellent inhibition of LBPs on the apoptosis of damage hepatocyte.

### 3.7 LBPs inhibited alcohol‐induced apoptotic pathways in L‐02 cells

**Figure 7:**
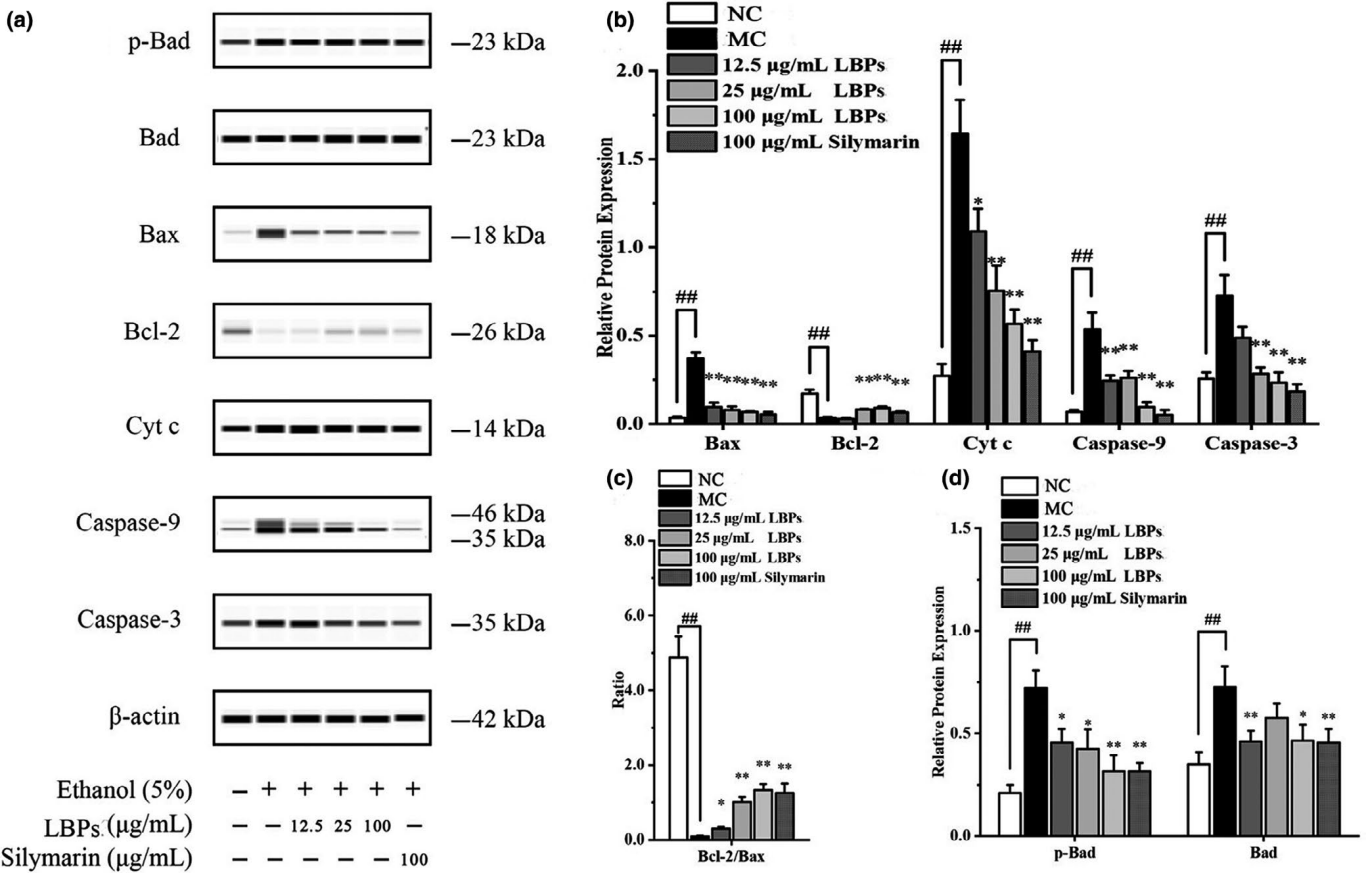
The effect of LBPs on the apoptosis‐provoking proteins in alcohol‐induced L‐02 cells (*n* = 3). (a) Western blot images on the apoptosis‐provoking proteins; (b) relative density analysis of the Bax, Bcl‐2, Cyt c, Caspase‐9, and Caspase‐3 protein bands; (c) relative density ratio of Bcl‐2 and Bax protein bands; (d) relative density analysis of the p‐Bad and Bad protein bands. Compared with the NC group *p < .05, **p < .01; compared with the MC group ^#^ *p* < .05, ^##^ *p* < .01

To observe whether the LBPs have the protective effects against ALD and possible mechanism related to apoptosis, Western blotting was applied to profile changes in expressions of related proteins. The suppression of MMP was related to a significant release of Cytochrome c (Cyto c) from mitochondria into cytoplasm. In Figure 7a,d, Cyto c was enhanced in cytoplasm of alcohol‐treated cells. Interestingly, treatment with LBPs showed dose‐dependent inhibition in this elevation. The increased amount of Cyto c in cytoplasm was associated with the activation of caspase‐3 and caspase‐9, the key “executer” protease of cell apoptosis. The levels of caspase‐3 and procaspase‐9 (47 kDa) in hepatocytes treated with alcohol were markedly enlarged (Figure 7a,b), whereas LBPs inhibited these effects. Moreover, only during apoptosis is procaspase‐9 (47 kDa) activated by proteolytic cleavage into its active subunits (35 kDa; Park et al., 2011). Consequently, the 35 kDa band of MC group was lighter than the other bands and gradually disappeared with the increase concentration of LBPs shown in Figure 7a. In addition, the expression of Bcl‐2 and Bax, apoptosis‐related protein, was also changed by alcohol, as compared with vehicle controls, as shown in Figure 7b. LBPs significantly restored the reduction of Bcl‐2 (antiapoptotic protein), increase of Bax (proapoptotic protein) in alcohol‐treated cells in a dose‐dependent manner, respectively. Accordingly, the ratio Bcl‐2/Bax rise markedly, with the dosage of LBPs increase (Figure 7c). We further investigated whether LBPs influenced Bad phosphorylation, basic operation for survival factors to block apoptosis. Figure 7d demonstrated that the expression levels of phosphorylated Bad were inhibited to 38.5% at the concentration of 100 μg/ml compared with model cells. This backs the argument that LBPs treatment effectively abolishes these alcohol‐induced proapoptotic events.

### 3.8 LBPs promoted the nuclear translocation of Nrf2 in L‐02 cells

**Figure 8:**
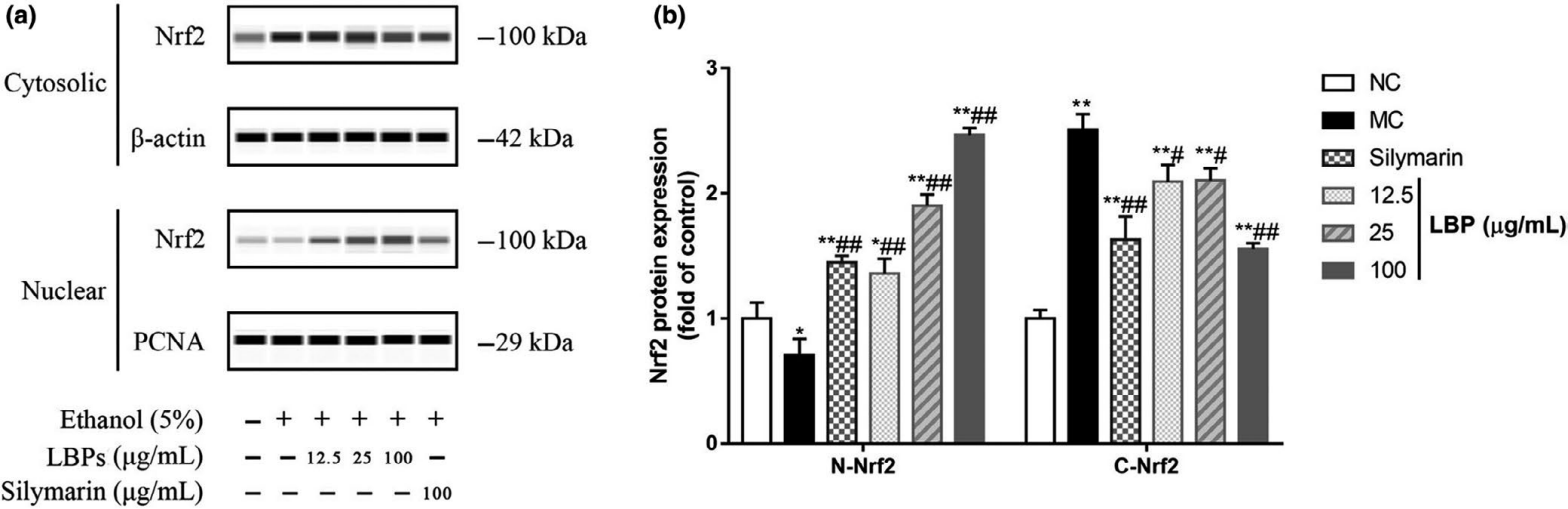
The effect of LBPs on the nuclear translocation of Nrf2 in alcohol‐treated L‐02 cells (*n* = 3). (a) Western blot images showing nuclear localization of Nrf2; (b) relative density analysis of the Nrf2 protein bands. Compared with the NC group *p < .05, **p < .01; compared with the MC group ^#^ *p* < .05, ^##^ *p* < .01

It has been documented that Nrf2 participates in protection against oxidative stress and vitality altered multiple signaling pathway. Thus, we further revealed the activation role of LBPs on the Nrf2 translocation path by Western blotting, as shown in Figure 8a. The relative density analysis in Figure 8b showed that, in the nuclear fraction of MC group, the expression of Nrf2 protein was reduced by 29.1% compared with normal cells (*p* < .01), while in LBPs/alcohol cotreated group the level of protein was significantly increased dose‐dependently. In contrast, LBPs intervention caused a dose‐dependent reduction of the amounts of cytosolic Nrf2, which were significantly elevated nearly 1.5‐fold in MC group (*p* < .01). LBPs activated Nrf2 through up‐regulating its protein expression and facilitating its nuclear translocation.

## 4 DISCUSSION

The present study investigated the possible mechanism by polysaccharides from *Lycium barbarum* L. exert their hepatoprotective effect on the oxidative damage and cell apoptosis induced by acute alcohol treatment of L‐02 cells.

The liver is the major organ in supporting the process of detoxification, lipid metabolism, where 90% of alcohol is metabolized. It is also the target for chemically induced injuries (Liu et al., 2012). The toxicity of the alcohol has been relatively well studied, particularly in the context of liver dysfunction and histopathological changes, including cell necrosis, cirrhosis, fibrosis, and life‐threatening (Mckillop & Schrum, 2005; Turati et al., 2014). In the present study, we established an acute alcoholic liver injury cell models in vitro to investigate the protective effects of LBPs. ALT and AST are very direct indicators to assess alcohol‐intoxicated hepatic injury (Gan et al., 2012). It was found that incubation of hepatic cells with alcohol led to acceleration of ALT and AST leakage and inhibition of LDH activities, which testified the availability and security of the model (Figure 1).

The disequilibrium between prooxidants and antioxidants in biological organism occurred in alcohol hepatotoxicity led to ROS production increases and lipid peroxidation occurs (Arteel, 2008; Liu et al., 2012; Tilg et al., 2011). ROS could destroy important structural and functional proteins in cells (Slater, 1984). The increased MDA, a particular biomarker of oxidative stress, indicates the enhancement of lipid peroxidation. There is a rationale certainly that antioxidant defense is a very important aspect for alleviating disease progression and even to halt it. LBPs exhibited powerful antioxidant and free radical scavenging activities of effective prevention on alcohol induced oxidative damage by down‐regulating ALT, AST, and LDH activities, scavenging the ROS and MDA. Moreover, the body responds to the overproduction of ROS and lipid peroxides by raising the expression levels of preventive antioxidants, including superoxide dismutase, catalase, and glutathione peroxidase. In aforementioned studies, the activities of these antioxidant enzymes in alcohol‐treated liver cells were evoked by LBPs. These alterations in liver enzymatic antioxidant defense further confirmed the induction of ROS and MDA following alcohol exposure. Therefore, treated with LBPs are indicative of enhanced oxidative insult, through improving the ability of antioxidant system.

Oxidative stress has been shown as initiator of apoptotic signaling pathway in cells (Chandra et al., 2000; Galán et al., 2001). Several lines of earlier investigations suggested that the generation of ROS through alcohol may act on mitochondria, resulting loss of MMP, modulating the functions of pro‐ and antiapoptotic proteins, eventually leading to cell death. Thus, further investigations were carried out to reveal the relationship between oxidative stress and apoptotic pathway induced by alcohol and underlying hepatoprotection mechanisms of LBPs. Mitochondria act as the sensor of oxidative stress. Alcohol caused a significant decrease in MMP (ψ_m_) as compared to MC group, signified the reinforcement of mitochondria dependent apoptosis involves outer membrane permeabilization. This process was controlled through up‐regulation of Bax (a proapoptosis factor) and down‐regulation of Bcl‐2 (an antiapoptosis factor), ultimately resulting in release of Cytochrome C and other proapoptotic factors from mitochondria into cytosol and initiated cell apoptosis (Herrera et al., 2010; Sinha et al., 2013). Moreover, the caspases that are among the main executors of the apoptotic process were triggered. Henceforth, the mitochondrial apoptotic pathway induced by alcohol was activated, ultimately leading to cell death. While, in the present study, we observed that LBPs down‐regulated proapoptotic Bax and up‐regulated antiapoptotic Bcl‐2 proteins, reversed MMP, enhanced the release of cytochrome C, activated caspase‐3 and 9 in L‐02 cells. From the above, LBPs can effectively inhibit all these alcohol‐mediated proapoptotic events. The results were also confirmed by apoptosis assay, which showed that LBPs treatment could reduce the DNA fragmentation and maintain the normal cellular structure and function against toxicity.

Previous studies demonstrated increasing Nrf‐2 activity is highly hepatoprotective during alcohol‐induced oxidative stress by mediating the response of the endogenous antioxidant system (Farombi et al., 2007; Yao et al., 2007). Nrf2 is considered as a pivotal regulator of oxidative stress in numerous cell types, taken part in the hepatoprotective effect of many naturally compounds (Jadeja et al., 2016; Zhang et al., 2015). All the results indicated LBPs could activate Nrf2 in L‐02 cells which could result in antiapoptosis via suppression of different downstream signal‐related incidents, including inhibiting of caspase‐3, modulating protein expression of Bcl‐2 family. However, the molecular signaling pathway responsible for the expression of Nrf2 was not determined and is worthy of further investigation.

## 5 CONCLUSION

In the light of the findings presented herein, natural product LBPs protects L‐02 cells from alcohol‐induced liver toxicity, oxidative stress, and apoptosis effectively. The antioxidant and antiapoptotic effect of LBPs is associated with its inhibition of the mitochondrial apoptotic pathway via Nrf2 signaling. Therefore, our study demonstrated that LBPs exerted promising hepatoprotective effect, which may have potential applications in prevention and protection of oxidative stress‐induced liver injuries.

## CONFLICT OF INTEREST

None.

## Data Availability

All data, models, and code generated or used during the study appear in the submitted article.
